# Effect of co-trimoxazole and N-acetylcysteine alone and in combination on bacterial adherence on ureteral stent surface

**DOI:** 10.1007/s00240-023-01508-5

**Published:** 2023-12-11

**Authors:** Mostafa AbdelRazek, Omar Mohamed, Reham Ashour, Mohamed Alemam, Mohamed El-Gelany, Mohammed S. Abdel-Kader

**Affiliations:** 1 Department of Urology, Qena University Hospital, Faculty of Medicine, South Valley University, P.O. Box 83523, Qena, Egypt; 2 Department of Clinical Pathology, Qena University Hospital, South Valley University, Qena, Egypt

**Keywords:** Ureteral stent, Bacterial adherence, Co-trimoxazole, N-acetylcysteine

## Abstract

To assess the effect of co-trimoxazole and N-acetylcysteine (NAC), alone and in combination, on bacterial adherence to biofilm formed on ureteral stent surfaces. This prospective randomized study was conducted on 636 patients who underwent double J ureteral stent insertion after variable urological procedures. Patients were randomized into four groups: A (*n* = 165), no antibiotics or mucolytics during stent indwelling; B (*n* = 153), oral NAC (200 mg/day for children aged < 12 years old and 600 mg/day for adults) during stent indwelling; C (*n* = 162), oral co-trimoxazole (2 mg TMP/kg/day) during stent indwelling; and D (*n* = 156), both oral NAC and co-trimoxazole during stent indwelling. Two weeks following double J stent (JJ stent) insertion, urinalysis was performed on all patients and urine culture was done for all the patients at the day of double J stent removal. The stent was removed 2 weeks postoperatively, and a stent segment sized 3–5 cm from the bladder segment of the stent was sent for culture. Positive stent cultures were found in 63.6% (105/165), 43.1% (66/153), 37% (60/162), and 19.2% (30/156) patients of groups A, B, C, and D, respectively. *E. coli* was the organism most commonly isolated from the stent culture in all groups. The combination of co-trimoxazole and NAC was more effective in reducing bacterial adherence on ureteral stent surfaces than either alone.

## Introduction

Ureteral stents are frequently utilized in urological practice [1]. The initial documentation of their use dates back to 1967 by Zimskind et al. [2]. These stents are employed after the treatment of renal and ureteric stones to alleviate kidneys obstructions, as well as following urological procedures, such as renal transplantation and ureteral stricture treatment [3]. Nevertheless, complications such as stent encrustation, blockage, displacement, and the development of biofilm and stone formations can occur [4].

Biofilm refers to a population of cells that grow on the surface of the stent and are encased in a matrix made of exopolysaccharides, which can ultimately lead to lumen blockage [5]. Biofilm formation occurs when bacteria transition from a free existence to firmly attaching themselves to the stent surface. This process involves the production of exopolysaccharide glycocalyx polymers, which form a matrix supporting the growth of microcolonies and the subsequent formation of biofilms [6].

Prior literature has indicated that approximately 65% of nosocomial infections are caused by biofilms formed by microorganisms. The increased use of stents has contributed to a rise in the incidence of complicated urinary tract infections (UTIs) [12]. Urinary stent-related infections can have severe consequences, including acute pyelonephritis, bacteremia, renal failure, and even death [13]. Co-trimoxazole, a combination of trimethoprim (TMP) and sulfamethoxazole, is known for its efficacy against common urinary tract pathogens, this antibiotic combination is commonly used to treat urinary tract infections, including those associated with urinary stents [7]. N-acetylcysteine (NAC) is a mucolytic agent that functions by disrupting disulfide bonds present in mucus. This action leads to a reduction in the viscosity of secretions [8]. It diminishes the production of extracellular polysaccharide matrix and mitigates biofilm formation [9]. Since the antimicrobial susceptibility of bacteria associated with biofilms is enhanced once the biofilm is disrupted [10], it is plausible that a combination of antibiofilm and antimicrobial agents would yield synergistic effects [11].

The objective of our study was to evaluate the impact of co-trimoxazole and N-acetylcysteine (NAC), both individually and in combination, on bacterial adherence bacterial adherence to biofilm formed on ureteral stent surfaces.

## Patients and methods

This study was a prospective randomized clinical trial conducted on patients who underwent double J ureteral stent insertion, specifically using polyurethane double J stents. The stents were left in place for a duration of 2 weeks, and the study was conducted between January 2017 and January 2020.

The sample size was determined using G*Power software (ver. 3.1.9.7; Heinrich-Heine-Universität Düsseldorf, Düsseldorf, Germany). A significance level (α) of 0.05 was chosen, corresponding to a 5% chance of making a Type I error. Statistical power (1—beta, β) used for the study was set at 80%, indicating the probability of detecting a true effect if it exists.

Eligible patients were randomized into four groups using a closed envelope method. Sequentially numbered closed opaque envelope used created by independent statistician and opened by pharmacist not in research team to ensure allocation concealment.

Group A, the control group, consisted of 165 patients who received no antibiotics or mucolytics, while the stent was indwelling (only receive placebo). Group B consisted of 153 patients receiving only oral NAC (200 mg/day for children less than 12 years of age and 600 mg/day for adults) alone, while the stent was indwelling. Group C consisted of 162 patients receiving only oral co-trimoxazole (2 mg TMP/kg/day, a prophylactic dose), while the stent was indwelling. Group D consisted of 156 patients receiving both oral NAC and co-trimoxazole, while the stent was indwelling with doses identical to groups B and C. Randomization was performed at our clinic. The protocol of this study was approved by the faculty ethical committee no UR0011-2-180. Informed consent, to the procedure and possible complications, was obtained from all patients. We excluded pregnant and lactating women, children less than 3 months of age, and all patients undergoing long term double J ureteral stent insertion, preoperative urinary tract infection (UTI), patients with anatomical urological abnormalities, urinary diversions, infection stones hypersensitivity to sulfa drugs, megaloblastic or folate deficiency anemia, significant renal or hepatic impairment, hypersensitivity to NAC, diabetics and immunocompromised patients and patients with acute asthma.

### Statistical analysis

The means with standard deviations (SDs) were used to record continuous variables, while frequencies were used to record categorical variables. Student *t* test was employed to assess differences between groups for continuous variables, while the Chi-square test was used for categorical variables. A *P* value of ≤ 0.05 was deemed statistically significant. All statistical analyses were conducted using IBM statistical software version 25 (Chicago, IL, USA).

**Fig. 1:**
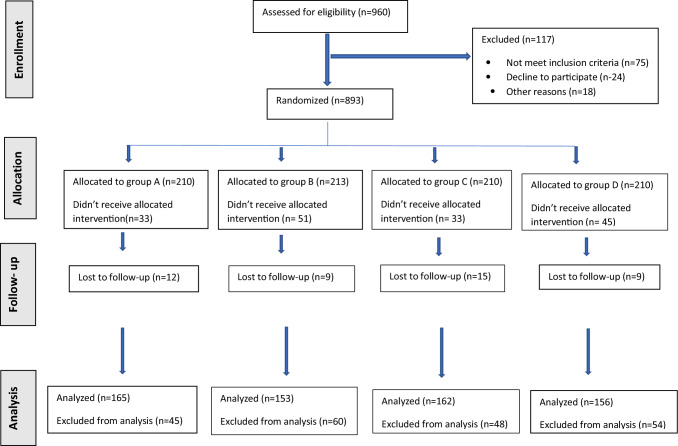
Consolidated Standard of Reporting Trials (CONSORT) diagram for patients flow throughout the study

Figure 1 illustrates the patient selection process throughout the study.

### Technique

All patients underwent preoperative clinical examination. Imaging included abdominopelvic ultrasound, KUB radiograph, and computed tomography without contrast. Laboratory studies included urinalysis and urine culture to exclude preoperative UTI. Following informed consent, a selective urine sample was obtained intraoperatively from the ureter using the ureteroscope before polyurethane JJ stent insertion. The urine sample was analyzed to exclude UTI and urine culture done.

Two weeks after stent insertion, urinalysis was performed for all patients and a urine culture was performed pre stent removal.

The stent was removed 2 weeks after insertion under aseptic conditions in the operating theater via cystoscope. A segment of the stent, about 3–5 cm from the bladder segment of the stent was placed in a sterile cup and sent to the laboratory for culture (Table 1).

**Table 1:** Indications for ureteral stent insertion

Indication	Number	A	B	C	D	*P* value
Ureteral stone (ureteroscopy)	282 (44.3%)	71	69	73	69	0.875
Ureteral stone (ureterolithotomy)	83 (13%)	22	20	20	21	0.984
Ureteral stricture (ureteroscopy)	58 (9.1%)	18	13	14	13	0.813
Ureteral stricture (reimplantation/Boari flab/resection–re-anastomosis)	42 (6.6%)	11	11	8	12	0.802
Pyeloplasty	61 (9.6%)	17	16	14	14	0.815
Renal stone (PNL)	37 (5.8%)	10	9	11	7	0.798
Pre-ESWL	73 (11.5%)	16	15	22	20	0.792

## Results

Patients’ ages are shown in Table 2. The mean ages were 41.73, 40.63, 39.52 and 44.25 years in groups A, B, C, and D, respectively, with no significant difference (*P* = 0.553). There was also no statistically significant difference in sex distribution among the groups (*P* = 0.673, Table 2).

**Table 2:** Patients’ demographics

Sex	Group A	Group B	Group C	Group D	*P* value
Number	165	153	162	156	
Age (year) Mean	41.73	40.63	39.52	44.25	0.553
Male:female	111:54	96:57	93:69	105:51	0.673
Size of stents used					
5–12	8	7	6	7	0.756
5–14	4	3	3	2	0.812
5–22	3	4	5	4	0.623
6–26	150	149	148	143	0.732

Two weeks following JJ stent insertion, urinalysis was performed on all patients. Significant pyuria was detected in 72.7% of group A, 68.6% of group B, 40.7% of group C, and 38.5% of group D. Table 2 notes the statistically significant differences in pyuria among the groups (*P* = 0.001) (Table 3).

**Table 3:** Two week post-operative urine analysis and culture

Organism	Group A	Group B	Group C	Group D	*P* value
Positive pyuria	120 (72.7%)	105 (68.6%)	66 (40.7%)	60 (38.5%)	0.001*
*E-coli*	35 (21%)	20 (13%)	13 (8%)	9 (5.8%)	0.0001*
*Klebsiella*	14 (8.5%)	5 (3.3%)	10 (6%)	2 (1.3%)	0.02*
*Pseudomonas*	8 (4.8%)	4 (2.6%)	5 (3%)	4 (2.6%)	0.6
*Staph*	3 (1.8%)	2 (1.3%)	0 (0%)	0 (0%)	0.2
*Candida*	4 (2.4%)	3 (2%)	2 (1.2%)	0 (0%)	0.3
Positive urine culture	64 (38.8%)	34(22.2%)	30 (18.5%)	15 (9.6%)	0.0001*
Negative urine culture	101 (61.2%)	119 (77.8%)	132 (81.5%)	141 (90.4%)	0.0001*

A urine culture was also performed for all patients. There was a highly significant difference observed among the groups (*P* = 0.0001) E. coli was found to be the most frequently isolated organism from the urine culture across all groups (Table 3).

Culture of the indwelling ureteral stents was positive in 63.6%, 43.1%, 37%, and 19.2% from groups A, B, C, and D, respectively. These differences were statistically significant (*P* = 0.001), as shown in Table 4. *E. coli* was most commonly isolated from the stent culture in each group.

**Table 4:** Bacteriology of double J ureteral stent culture

Organism	Group A	Group B	Group C	Group D	*P* value
*E-coli*	57 (34.5%)	39 (25.4%)	24 (14.7%)	18 (11.5%)	0.015*
*Klebsiella*	24 (14.6%)	9 (5.9%)	21 (13%)	3 (1.9%)	0.048*
*Pseudomonas*	12 (7.3%)	9 (5.9%)	9 (5.6%)	9 (5.8%)	0.983
*Staph*	6 (3.6%)	3 (2%)	0 (0%)	0 (0%)	0.214
*Candida*	6 (3.6%)	6 (3.9%)	6 (3.7%)	0 (0%)	0.329
Positive stent culture	105 (63.6%)	66 (43.1%)	60 (37%)	30 (19.2%)	0.001*
Negative stent culture	60 (36.4%)	87 (56.9%)	102 (63%)	126 (80.8%)	0.001*

Adverse reaction of drugs of each group tabulated in Table 5

**Table 5:** Adverse reaction of drugs used in each group

Adverse reaction	Group A	Group B	Group C	Group D
Nausea	0	2	7	5
Vomiting	1	2	3	4
Rash	0	0	1	1
Itching	0	0	4	2
Sore throat	0	0	0	0
Fever or chills	0	0	0	0
Diarrhea	0	0	2	1
Cough	0	0	0	0
Joint or muscle pain	0	0	1	2

## Discussion

The utilization of ureteral stents in urological practice has seen a notable increase in recent years. Unfortunately, the presence of these stents contributes to the formation of biofilms. Extensive research has demonstrated that biofilms, primarily formed by microorganisms, are responsible for approximately 65% of nosocomial infections. As the use of stents continues to rise, so does the incidence of complicated urinary tract infections (UTIs) [12]. Infections linked to urinary stents can lead to substantial morbidity, including bacteremia, acute pyelonephritis, renal failure, and may ends to mortality [13].

Bacterial colonization of the stent plays a crucial role in the development of stent-related infections [14]. Moreover, biofilms serve as sources of infection in other areas of the body through biofilm sloughing and bacterial detachment [15]. Consequently, preventing bacterial adherence to indwelling catheters or host cells themselves may reduce the incidence of biofilm-associated infections [16].

N-acetylcysteine (NAC) is a widely used medication for the treatment of paracetamol overdose and as a compound that helps to break down mucus. It has a well-established safety record and is approved for use in conditions characterized by abnormal, thick, or sticky mucus secretions, such as pneumonia, bronchitis, tracheobronchitis, cystic fibrosis, patients with tracheostomies, postoperative pulmonary complications, post-traumatic chest conditions, and as a preparation before diagnostic bronchoscopy to alleviate mucus blockage. NAC replenishes glutathione reserves by providing cysteine, an essential building block for glutathione production. In addition, NAC has the ability to bind to toxic metabolites and scavenge free radicals. It also enhances oxygen delivery to tissues, increases the production of mitochondrial ATP, and modulates microvascular tone to improve blood flow and oxygen supply to the liver and other vital organs.

In a study by Pérez-Giraldo et al., it was reported that N-acetylcysteine, protamine sulfate, Bandazac lysine, and sodium salicylate demonstrated the ability to reduce or hinder bacterial adherence and disrupt established biofilms [9]. Another study by Reid et al. involved the oral administration of ofloxacin and ciprofloxacin to forty patients with ureteric stents. The results showed that the drug levels on the surfaces of the devices were higher than the minimum inhibitory concentration (MIC) required to inhibit the growth of *E. coli*, *E. faecalis*, *P. aeruginosa* and *S. aureus*. In addition, no bacteria were detected in the patients’ urine samples, and no biofilms were observed [17]

No clinical studies have been published discussing the effect of the combination of NAC and an antibiotic on biofilm formation and bacterial adherence on ureteral stent surfaces. However, El-Feky et al. and El-Rehewy et al. in 2009 published in vitro studies on the effect of ciprofloxacin and NAC on bacterial adherence and biofilm formation [18, 19].

Our study is an in vivo study comparing the effect of oral NAC and co-trimoxazole alone and in combination on bacterial adherence and biofilm formation. Co-trimoxazole was chosen as the antibiotic of choice due to its composition of trimethoprim (TMP) and sulfamethoxazole. These two drugs target different steps in the pathway of an essential enzymatic reaction in bacteria. As a result, their combined action exhibits a synergistic effect, enhancing their effectiveness against bacterial infections [20].

After 2 weeks of treatment, urinalysis revealed that 72.7% of the control group had significant pyuria, which matches the results of Pooli et al. [21]. This rate decreased to 68.6% with the use of NAC and to 40.7% with the use of co-trimoxazole. Combination therapy decreased the rate of pyuria to 38.5% which was quite significant (*P* = 0.001).

Urine culture of the patients revealed that 38.8% of the control group were positive. This poor correlation between positive findings on urinalysis and positive urine culture in patients with indwelling stents suggests that urine culture is necessary to diagnose UTI. This also matches the results of Pooli et al. [21]. With the use of oral N-acetylcysteine, this rate of positive urine cultures among patients with pyuria decreased to 22.2% and decreased to 18.5% with the use of oral co-trimoxazole. With combination therapy, the rate of positive urine culture decreased to 9.6%. Which was statistically significant difference in the rate of positive urine culture after the use of NAC or co-trimoxazole (*P* = 0.0001), either alone or in combination. E. coli was the organism most commonly isolated from culture in each group.

Regarding stent culture Positive stent cultures were found in 63.6% (105/165), 43.1% (66/153), 37% (60/162), and 19.2% (30/156) patients of groups A, B, C, and D, respectively. close to the results of El-Feky et al. and El-Rehewy et al. [18, 19]. In all groups, the most frequently isolated organism from the stent culture was Escherichia coli (E. coli), followed by Klebsiella and Pseudomonas. These findings align with the results reported by Kehinde et al., demonstrating similar trends in the prevalence of these bacterial species in stent-associated infections [22].

In our study, a distinction exists between urine culture and stent culture positivity. Our study revealed that the rate of positive stent culture was higher than that of positive urine culture across the groups. In addition, all patients with positive urine culture also had positive stent cultures. Among the patients with positive urine culture, we observed that the type of bacteria identified was consistent with the bacteria found in the stent culture. Furthermore, in certain cases, we identified different types of bacterial colonies on the stents compared to those found in the urine culture of the same patient, although all the bacteria types detected in the urine culture were also present in the stent culture.

Adverse reaction of drugs of each group was mild and self-limited as hypersensitive patients to drugs used was excluded from the study (Table 5).

While both oral NAC and oral co-trimoxazole can have a role in reducing bacterial adherence and biofilm formation on ureteral stent surfaces, the combination of both has a greater effect on reducing bacterial adherence and biofilm formation on these surfaces.

### Study limitations

The study had several limitations, one of which was the relatively small number of patients included. This limited sample size may have increased the potential for statistical error during the analysis. Various patients co morbidities and its relation to increase incidence of bacterial adherence not analyzed but major co-morbidities that affect incidence of UTI-like diabetic patient were excluded.
